# Identification of biomarker candidates for exfoliative glaucoma from autoimmunity profiling

**DOI:** 10.1186/s12886-024-03314-y

**Published:** 2024-01-29

**Authors:** Ryan Potter, Marcelo Ayala, Andreas Tilevik

**Affiliations:** 1 Systems Biology Research Centre, School of Bioscience, University of Skövde, Skövde, Sweden; 2 Skaraborgs Sjukhus, Skövde, Sweden; 3 Karolinska Institutet: Stockholm, Stockholm, Sweden; 4 Sahlgrenska Academy, Gothenburg University, Gothenburg, Sweden

**Keywords:** Glaucoma, Exfoliation, Biomarkers, Autoimmunity, Genes

## Abstract

**Background:**

Exfoliative glaucoma (XFG) is a subtype of open-angle glaucoma characterized by distinctive extracellular fibrils and a yet unknown pathogenesis potentially involving immune-related factors. The aim of this exploratory study was to identify biomarkers for XFG using data from autoimmunity profiling performed on blood samples from a Scandinavian cohort of patients.

**Methods:**

Autoantibody screening was analyzed against 258 different protein fragments in blood samples taken from 30 patients diagnosed with XFG and 30 healthy donors. The 258 protein fragments were selected based on a preliminary study performed on 3072 randomly selected antigens and antigens associated with the eye. The “limma” package was used to perform moderated t-tests on the proteomic data to identify differentially expressed reactivity between the groups.

**Results:**

Multiple associated genes were highlighted as possible biomarker candidates including FUT2, CDH5, and the LOX family genes. Using seven variables, our binary logistic regression model was able to classify the cases from the controls with an AUC of 0.85, and our reduced model using only one variable corresponding to the FUT2 gene provided an AUC of 0.75, based on LOOCV. Furthermore, over-representation gene analysis was performed to identify pathways that were associated with antigens differentially bound to self-antibodies. This highlighted the enrichment of pathways related to collagen fibril formation and the regulatory molecules mir-3176 and mir-876-5p.

**Conclusions:**

This study suggests several potential biomarkers that may be useful in developing further models of the pathology of XFG. In particular, *CDH5, FUT2*, and the *LOX* family seem to have a relationship which merits additional exploration.

**Supplementary Information:**

The online version contains supplementary material available at 10.1186/s12886-024-03314-y.

## Introduction

Glaucoma categorizes a set of eye diseases that collectively represent the leading cause of irreversible blindness globally, affecting nearly 70 million individuals worldwide [1]. Increasing evidence points to autoimmunity as a contributor to the retinal neurodegeneration associated with this disease [2–5]. It is estimated that as much as 43% of patients who have glaucoma remain undiagnosed, with this undiagnosed population being mostly individuals under age 60 [6]. This indicates that methods to detect the disease earlier may have a massive impact on the well-being of a substantial subset of the population.

Exfoliation syndrome (XFS) is a leading cause of open-angle glaucoma, especially in the Scandinavian population, resulting in a subtype known as exfoliation glaucoma (XFG) [7]. Several genetic and non-genetic risk factors have been identified for XFG. However, the molecular mechanism of deposit formation and how that contributes to the onset of glaucoma is yet unexplained [8]. XFG is characterized by elevated intraocular pressure (IOP) and white flakes accumulating in the anterior lens of the eye and is thought to be associated with cataract formation [9]. Autoimmunity describes any immune response that reacts with a self-antigen, i.e., molecules produced as a normal component of the organism in which the response occurs [10]. Autoantibodies are not only present in individuals with autoimmune diseases but can also be found in healthy individuals [11]. These natural autoantibodies, primarily IgM, play important roles in the immune system, such as providing a first line of defense against infections and contributing to immune system homeostasis [12]. In contrast, high-affinity IgG autoantibodies often reflect underlying pathological processes and can serve as biomarkers for autoimmune disorders [12]. A recent meta-analysis identified 77 autoantibodies that are commonly expressed in healthy individuals, including 20 which have been previously identified as potential disease biomarkers [13]. Autoantibodies in healthy individuals may develop due to, for example, similarity to common viral proteins. There has been much interest in identifying to what extent autoimmune activity may be implicated in glaucoma patients, and many differing results have been suggested between the various disease subtypes [14, 15]. Despite this, little consensus exists regarding which specific mechanisms motivate this, or if all subtypes share any common autoimmune action [16].

By developing a profile of which proteins are implicated in the pathogenesis of a given autoimmune condition, a biomarker model may be constructed to enable the detection of the condition and monitor response to treatments [17]. Furthermore, understanding the targets of this renegade autoimmunity in certain diseases has allowed for the development of complex biologic therapies, often through antibodies which inhibit B- or T- cells within the immune system [18].

The lysyl oxidase (*LOX*) family of genes has been identified by several studies as of particular interest as a potential biomarker related to XFG [7, 19–21]. These genes, especially *LOXL1*, are believed to synthesize and maintain elastic tissues, which have been found to be increased in the early stages of XFG progression [7]. Further evidence indicates that vesicular transport of the LOXL1 proteins to the surface of the cell membrane may allow the proteins to participate in XFS fibrils [21]. Schlötzer-Schrehardt suggests that the presentation of LOXL1 proteins on the surface, in parallel with the expression of binding partners (e.g. tropoelastin, fibulin-5, etc.), leads to an aggregation of abnormal XFS fibrils [21]. In 2022, a study by Ayala et al., investigated the association between two single nucleotide polymorphisms (SNPs) in the *LOXL1* gene and the progression of exfoliation glaucoma in Swedish patients [20]. The study enrolled 130 patients with exfoliation glaucoma and assessed their glaucoma progression using various measures. The results demonstrated a significant association between the SNPs LOXL1_rs2165241 and LOXL1_rs1048661 and the progression of XFG. This was the first study to show such an association, suggesting that these SNPs could potentially serve as biomarkers for early detection and intervention in patients with XFG.

Given its critical role in the structural integrity of extracellular matrices, any aberrations in the *LOXL1* gene could potentially lead to abnormal protein structures or expressions. Such alterations are pivotal as they might render the protein more immunogenic, becoming potential targets for autoantibodies [22]. In the context of our study, antibody profiling against proteins, including those derived from *LOXL1*, is crucial. It allows us to discern if there is an autoimmune component in XFG patients targeting these proteins, thereby providing a deeper understanding of the gene’s functional implications in XFG pathogenesis. Moreover, profiling antibodies against antigens associated with such crucial genes aids in mapping out the intricate interplay between genetic predispositions and immune responses in XFG.

The aim of the present study was to identify potential serum-based biomarkers for XFG patients.

## Materials and methods

### Patients

The present study was a case-control study. At inclusion, all subjects signed written consent. The study followed the tenets of the Declaration of Helsinki. All cases were diagnosed with XFG. The controls were healthy individuals. XFG patients were recruited among glaucoma patients coming to the Eye Department. The healthy individuals were recruited among blood donors. XFG was defined based on the European Glaucoma Society guidelines [23]. The healthy individuals were subjects with no known eye diseases, which was reconfirmed through an ophthalmic examination (see below). All included subjects were recruited at the Eye Department, Skaraborg’s Hospital, Västra Götaland Region (VGR). The inclusion period was from 1st January, 2014, to 31st December, 2018. At inclusion, both patients and healthy individuals answered a questionnaire regarding general diseases like hypertension, diabetes, smoking, autoimmune disease, etc. (see supplementary materials). In the case patients or controls reported a previous diagnosis of an autoimmune disease, they were excluded from the study. After this, individuals were provided a full ophthalmological exam by the same ophthalmologist (MA). The visual acuity was measured using a Snellen’s table and was determined in the range 0–1. The examination included best corrected visual acuity (BCVA) and intraocular pressure (IOP) measurements with a Goldmann’s applanation tonometer. Three measurements were done, and the mean value was recorded. Afterwards, a visual field test using a Humphrey device (Carl Zeiss, Carl-Zeiss-Straße 22, 73,447 Oberkochen, Germany) was performed by an ophthalmic nurse. The strategy used for visual field testing was the 24 − 2 fast program. Then, a gonioscopy was performed using a goniolens in a dark room to assess the trabecular meshwork. Then the pupils were dilated. The anterior chamber was analyzed using a slit-lamp. The presence or absence of exfoliation was recorded. The optic nerve was assessed using a 90-D lens. In the case of glaucoma patients, the number of medicines was registered. After the ophthalmic examination, blood samples were taken. The blood samples were centrifugated and the serum was stored in a freezer at -80 C. The serum samples were then transported on dry ice to SciLifeLab, Stockholm, Sweden, for analysis. Statistical tests comparing the baseline characteristics between the healthy individuals and XFG patients, were carried out by an unpaired t-test for variables on continuous scale, and by a chi-square test for variables on a categorical scale. All statistical tests were two-sided and p-values less than 0.05 were considered significant.

### Preliminary study

A preliminary study was conducted using serum samples of 30 randomly chosen patients with XFG and ten control samples from anonymous blood donors included in the SciLifeLab’s Global Immunodeficiency Project in order to identify a set of candidate autoantigens. This study examined the binding of antibodies against a total of 3072 different antigens associated with genes expressed in the eye and retina, as well as randomly selected from the Human Protein Atlas. The serum samples were diluted 1:250 in assay buffer (0.1% PBS-Tween20, 3% BSA, 5% milk, supplemented with 160ug/ml His6ABP), incubated in assay buffer for 30 min, and then transferred to the slides containing the antigen arrays. The samples were incubated on the arrays for 1 h at room temperature on a shaker (240 rpm). After washing in 0.01% PBS-Tween20, the slides were then incubated with hen anti-His6ABP (produced within The Human Protein Atlas Project) for 1 h on a shaker. The secondary anti-human IgG, Fcγ specific, conjugated with Alexa 647 (Jackson Immunoresearch Cat. #109-606-008, 2 mg/ml) with dilution 1:25,000 and Goat anti-chicken IgY Alexa 555 (Invitrogen Cat. #A-21,437) with dilution 1:60,000 were used with 1 h incubation at room temperature. After washing in 0.01% PBS-Tween20, the slides were then scanned using a LuxScan™ HT24 Microarray scanner (CapitalBio Corporation) at a resolution of 10 μm. The images from the scanner were analyzed using GenePix Pro 5.1 image analysis program. R was used for the statistical analysis. The result from the image analysis was filtered for features with less than 30 pixels in size or flagged during image analysis. Subsequently, features with at least 25% (*N* = 10) of the samples with NA were excluded from the data (*N* = 104), and for replicate antigens only the array batch with the highest mean green signal (printing control) for the antigen was included (*N* = 12 features excluded). The remaining data was transformed per block to the number of median absolute deviations (MADs) around the median in order to adjust for batch and sample specific backgrounds. A cutoff of 70 MADs above the median was applied to transform the data into binary data (reactive vs. not reactive). We identified 60 different antigens that showed reactivity against autoantibodies in at least three samples (see supplementary materials).

### Suspension bead array

To verify the antibody binding to the 60 reactive autoantigens found in the preliminary study, as well as an addition of 45 antigens from a literature search for genes associated with eye and eye diseases, a total of 105 different antigens were further analyzed with a Luminex assay. This study involved the same 30 XFG patients as in the preliminary study but replaced the ten anonymous blood samples with new age-matched controls (*n* = 30) as previously described (see Patients). The reason for recruiting new controls was to collect the same number of controls as patients, as well as to get a better age-match between the two groups. On average, each antigen was analyzed based on about two different antigen fragments. In total, 258 protein fragments were therefore analyzed. The 258 protein fragments, plus 4 controls, were immobilized on color coded magnetic beads (MagPlex, Luminex Corp., Austin, TX) using COOH-NH2 chemistry. The antigen fragments were coupled into beads in 3 separate 96-well plates. The included controls consisted of His6ABP (control of binding to protein fragment tag, HPA resource), buffer blank (control of binding to bare bead), rabbit anti-human IgG (loading control, 309-005-082, Jackson immunoresearch), and Epstein-Barr nuclear antigen 1 (EBNA1, positive control, high frequency expected, ab138345, abcam). The coupling efficiency was tested using antibodies towards His6ABP (HPA resource). The serum samples were diluted 1:250 in assay buffer (3% BSA and 5% milk in PBS supplemented with 0.05% Tween-20 and 160 µg/ml His6ABP tag). Commercial plasma (PK2-123-v-37,973 Human plasma EDTA K2 mixed 50% male and female, Seralab), included as technical controls, were diluted similarly to the samples in 3 wells, and 3 buffer-wells were included as negative controls. The diluted samples were incubated for 1 h at room temperature for pre-blocking of any antibodies toward the albumin binding protein domain before mixing with the antigen bead array, as the albumin binding protein domain is derived from gram-positive bacteria. Subsequently, the diluted samples were incubated with the antigen bead array for 2 h. The bound antibodies were thereafter fixated using 0.02% paraformaldehyde (43368-9 M, Alfa Aesar) for 10 min. Goat fab fragments anti-human IgG Fγ labeled with R-phycoerythrin (Cat#12-49998-82, eBioscience) were then applied for 30 min to enable a read-out using a FlexMap 3D instrument (Luminex Corp., Austin, TX). The read-out consists of the median fluorescent intensity (MFI) and the number of beads the median is based on for each antigen (bead ID) in each sample. For quality control, both MFI and bead count data were used to exclude any antigen fragments and samples that did not meet the technical criteria at SciLifeLab.

### Pre-processing

A baseline for background noise was first established by determining outliers within the readings for empty wells across the non-control analytes measured. Empty-well outlier threshold was determined as any analyte wherein the mean of all empty-well sample measurements was greater than one standard deviation from the median value of all analytes’ means of empty-well samples. Maximum value plus one standard deviation for the remaining empty-well readings was selected as the assumed noise threshold for each of the two technical replicates. Any analytes that had no readings above these cutoff values were excluded from further analysis within each dataset. The MFI values were averaged between readings for each patient/analyte of the two technical replicates. Readings were validated for similarity between technical replicates to identify any single anomalous data points. The average value of all empty wells for each analyte was subtracted across samples. To stabilize the variance and normalize the data, we applied Weighted Box-Cox (wBC) and Robust Spline Normalization (RSN) transformations to the dataset prior to downstream analysis.

### Differential antibody reactivity

Differential expression analysis was performed using the “limma” package in R (version 3.54.2). Limma’s empirical Bayes methods have been demonstrated as a powerful tool, which outperforms classical statistical tests in classifying protein expression data [24]. Importantly, the wBC transformation was applied to ensure that the data met Limma’s assumption of a common mean-variance relationship across antibodies. Adjusted p-values were simultaneously calculated with the limma package using the Benjamini-Hochberg method.

### Heatmap and PCA

To visualize the general ability to cluster diseased patients from the healthy ones, both a heatmap and principal component analysis (PCA) plot were constructed. The PCA plot includes data across all analytes using singular value decomposition methodology via the prcomp function in R. The heatmap was constructed using the pheatmap package in R. Hierarchical clustering was applied to the columns and rows of the heatmap, which groups similar patients and analytes based on the pairwise Euclidean distance.

### Classification

To analyze the possibility to discriminate patients with XFG from the healthy controls, a logistic regression model was used. The model was trained using analytes with an adjusted p-value below 0.05. The resulting logistic regression equation was used to compute the probability of a sample belonging to the diseased group. Backward step elimination was used to reduce the model based on the Akaike information criterion (AIC), and the models were validated using leave-one-out cross-validation (LOOCV). ROC curves were constructed to evaluate the performance of the two logistic regression models in predicting the disease state (healthy vs. diseased).

### Gene set analysis

In our study, we sought to identify proteins that are targeted by autoantibodies in XFG patients. While autoantibodies directly target proteins, these proteins are encoded by genes. To further understand the biological relationship between the proteins, we employed over-representation analysis (ORA) by comparing a set of genes associated with the most significant analytes in this study against standard gene sets. Since ORA requires at least around 20 genes to be meaningful to run, we broadened our set for this test to include those significant prior to correction.

The list of genes associated with analytes with significant (*p* < 0.05) raw p-values from the limma analysis were analyzed using ORA against the C2, and C3 gene sets hosted in the Broad Institute’s Molecular Signatures Database (MSigDB) [25, 26]. The MSigDB is a collection of annotated gene sets for use with gene set enrichment analysis and other gene set analysis tools. The C2 curated gene set was selected as an initial reference due to its broad representation of many databases representing canonical pathways and the C3 regulatory target gene sets were selected for investigating the regulatory relationships in biological processes and their potential impact on gene expression changes.

## Results

### Patients

In total, sixty individuals were recruited for this study. Thirty were diagnosed with XFG and thirty were healthy (Table 1). Among the XFG patients, twenty-five (83%) showed unilateral glaucoma. Regarding the amount of IOP-reducing eyedrops in the XFG patients, the average was 2.44 (SD: 0.77). No eyedrops were used in the healthy group. The age of the XFG patients was a bit higher than the healthy individuals, but the difference was not statistically significant (*p* = 0.07). As expected, IOP and visual field parameters differed significantly between XFG patients and healthy subjects (*p* < 0.001).

**Table 1:** Baseline characteristics of the subjects

	Healthy individuals (*N* = 30)	XFG patients (*N* = 30)	p-value
Age (years) (SD)	70.93	73.16	0.07
Sex (F/M) (%)	16/14 (53/47)	13/17 (43/57)	0.43
Diabetes (Yes/No) (%)	3/27 (10/90)	5/25 (17/83)	0.45
Hypertension (Yes/No) (%)	4/26 (13/87)	6/24 (20/80)	0.49
Smoking (Yes/No) (%)	1/29 (3/97)	3/27 (10/90)	0.30
BCVA (SD)	0.95 (0.08)	0.81 (0.19)	0.007
IOP (mmHg) (SD)	15.53 (1.35)	20.42 (1.42)	< 0.001
MD (dB) (SD)	-0.38 (0.54)	-7.87 (4.68)	< 0.001
VFI (%) (SD)	97.43 (1.35)	82.33 (13.55)	< 0.001

F: Female. M: Male. BCVA: Best corrected visual acuity. IOP: Intraocular pressure. MD: Mean deviation. VFI: Visual field index

### Differential antibody reactivity

Out of the initial 3072 antigens, our preliminary study using protein microarray identified 60 antigens with a reactivity cutoff point of > 70 Median Absolute Deviations (MADs) in at least three serum samples (see supplementary data). In addition to these 60 antigens, 45 antigens from a literature search for genes associated with eye and eye diseases were included in the Luminex assay. Because many of the antibodies targeted correspond with multiple antigen fragments, approximately two fragments were selected for each target, on average. Additionally, many of the fragments correspond to multiple gene targets, so in total, 137 related genes associated with 258 protein fragments were analyzed. Two technical replicates were run. Prior to merging the two technical replicates, two analytes were identified as being below the background noise levels in both replicates and were thus removed from further analysis. In total, 256 analytes were therefore included in our aggregate dataset. Statistical tests using the “limma” package resulted in seven antigens with an adjusted p-value below 0.05 (DGCR2, LOX, FUT2, LGSN, ANXA10, TMEM9B, CDH5) (Table 2 and supplementary Fig. 1). In order to better depict the biological implication, fold-change values were obtained from the non-transformed dataset. The greatest changes in total expression were associated with CDH5 with a fold-change level of 2.16.

**Table 2:** Differentially bound antigen fragments and associated genes (adj. *p* < 0.05)

Antigen fragment	p-value	adj p-value	log2FC	FC	Gene name
HPRA000767	< 0.0001	0.0016	0.4404	1.3570	DGCR2
HPRA034083	< 0.0001	0.0016	0.6111	1.5274	LOX
HPRA006876	< 0.0001	0.0040	0.4992	1.4135	FUT2
HPRA019035	0.0001	0.0068	0.7008	1.6254	LGSN
HPRA003490	0.0003	0.0178	0.3633	1.2863	ANXA10
HPRA022019	0.0005	0.0241	-0.8738	0.5459	TMEM9B
HPRA017192	0.0006	0.0253	1.1109	2.1598	CDH5

### Heatmap and PCA

**Fig. 1:**
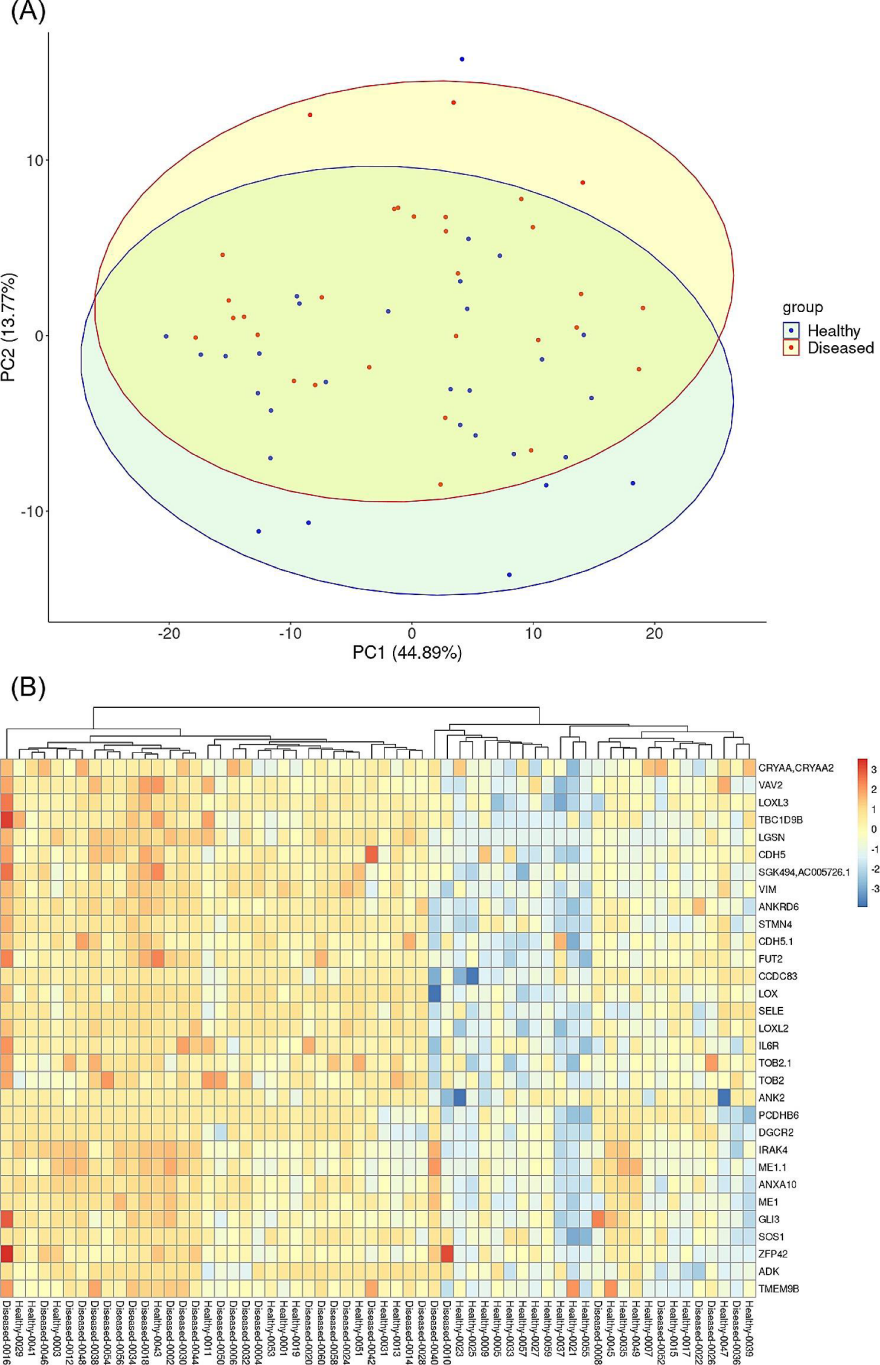
(**A**) Principal component analysis (PCA) of wBC/RSN transformed MFI data across all analytes. The x-axis represents PC1 which accounts for 44.89% of the total variation, while the y-axis represents PC2, accounting for 13.77% of the variation. The data points are colored according to their group, while the 95% concentration ellipses are shown to aid in the interpretation of the groupings. (**B**) Heatmap showing wBC/RSN transformed MFI levels across analytes identified as significantly different between healthy and patient groups based on raw p-value < 0.05. Columns were standardized and then both rows and columns were clustered based on complete linkage using Euclidean distance

In order to visualize the general ability to cluster diseased patients from the healthy ones, both a heatmap and principal component analysis plot were constructed (Fig. 1). The first principal component accounted for 44.89% of the variance observed in the dataset, while the second principal component accounted for 13.77% of the variance. Colored ellipses highlight the 95% concentration ellipse for either group. While there is some ability to distinguish the groups, there is a substantial overlap. This is supported in the heatmap (Fig. 1B), which focuses on all analytes with a raw p-value less than 0.05, based on the limma analysis. In the heatmap, red color represents higher MFI levels of a given analyte, while blue corresponds to low MFI. No clear separation of the patients and the healthy controls could be found although one small cluster with mainly healthy controls was found to have a generally low reactivity across the significant antigens.

### Classification

A logistic regression model was constructed using the seven analytes (Table 2) to classify healthy and diseased patients. The fitted regression model is shown below:

$$\eqalign{  {\text{logit}}\left( {\text{p}} \right) &  =  - 51.499 - 89.982\,*\,{\text{HPRA}}000767 - 140.131\,*\,{\text{HPRA0}}34083\\ &\quad + 1051.263\,*\,{\text{HPRA}}006876 + 51.803\,*\,{\text{HPRA}}019035\\ &\quad + 149.026\,*\,{\text{HPRA}}003490 - 10.914\,*\,{\text{HPRA}}022019\\ &\quad + 112.818\,*\,{\text{HPRA}}017192 \\} $$

After backward stepwise reduction, only a single protein fragment, associated with the gene FUT2, was retained in the reduced model:

$${\text{logit}}\left( {\text{p}} \right) =  - 58.695 + 1279.862\,*\,{\text{HPRA}}006876$$

**Fig. 2:**
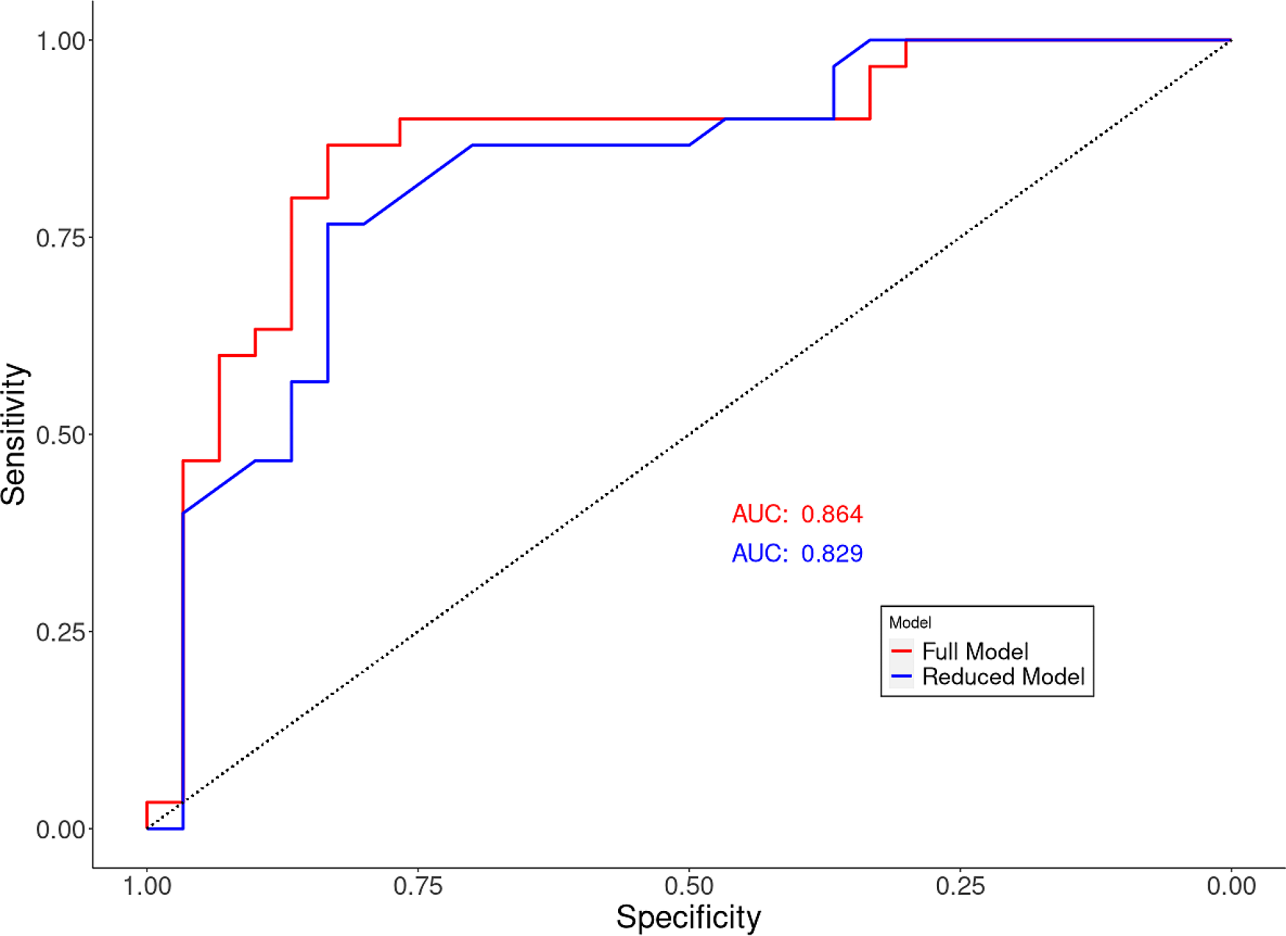
ROC curve based on logistic regression on analytes with an adjusted p-value below 0.05 (red) and based on the reduced model including only a single analyte (blue)

The area under the ROC curves (Fig. 2) was 0.864 for the model including seven protein fragments, and 0.829 for the reduced model. Both models were validated with LOOCV, yielding accuracies of 0.85 and 0.75, respectively.

### Gene set analysis

**Fig. 3:**
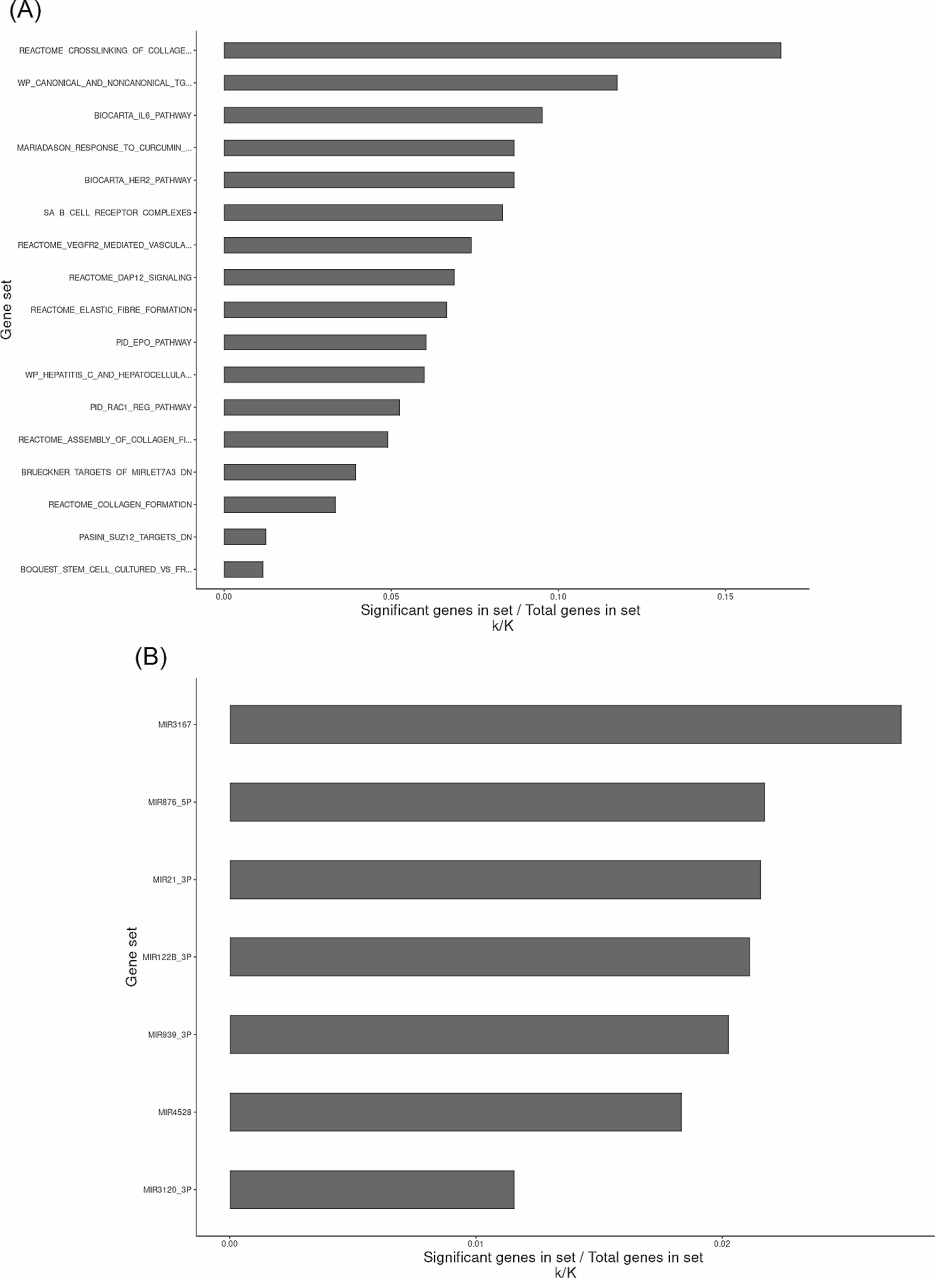
(**A**) Bar chart showing significantly (adj. p < 0.05) enriched gene sets in a list of genes associated with differentially expressed analytes, as compared against MSigDB C2 gene sets, sorted by ratio of significant over total genes in set (k/K). Several pathways identified as enriched correspond to collagen formation, a process hypothesized to be related to the pathogenesis of XFG. (**B**) Bar chart showing significantly (adj. p < 0.05) enriched gene sets, as compared against MsigDB C3 gene sets. Targets of miRNAs 3167 and 876-5P are the highest enrichment among the regulatory set, and contain many of the same genes implicated in the C2 gene sets

To understand the relationship between the proteins targeted by the autoantibodies, a gene set analysis was performed. The ORA showed significant (adj *P* < 0.05) enrichment across a total of 17 classifications (Fig. 3A). The most enriched pathway in the significant genes compared with the C2 gene set was related to the crosslinking of collagen fibrils. Furthermore, the same list was analyzed against C3 regulatory gene set. Within the C3 ORA, seven potential targets of regulatory function were identified as significantly (adj. *P* < 0.05) enriched (Fig. 3B). The top two categorizations correspond to the sets of gene targets of miR3167 and miR876-5P. These two sets are highly similar and contain 109 genes in overlap out of 110 in total in the miR3167-related gene set and 138 in the miR876-5P-related set. Genes that contributed to the enrichment of collagen-related pathways such as *LOX*, *VIM*, and *ANK2* are included in the targets of these miRNAs.

## Discussion

This study identifies several autoantibodies which are differentially reactive in XFG patients compared with non-XFG controls, allowing for a classification model with reasonably high accuracy. Some of these autoantibodies react with proteins which have previously been associated with glaucoma, such as LOX and TMEM9B, as well as proteins which have little or no previous association with the disease, such as CDH5 and FUT2.

Glaucoma has been associated with autoimmune activity since the early 1990’s [27]. Despite this, the mechanisms which lead to the condition are scarcely described and generally lack consensus across studies. Given the variety of potentially different pathologies that are categorized as glaucoma subtypes, it might be that similarities in these disease variants lead to misclassification, thereby obfuscating patterns that might otherwise be unique between them. This is further complicated by the fact that autoantibodies are regularly observed in widely varying amounts across healthy patient populations [28].

In addition to our study, a similar study was conducted using a protein macroarray that highlighted several autoantigens which showed increased presence in XFG patients compared to controls [29]. Several of the antigens identified by Dervan et al. were related to transmembrane proteins, leading to speculation that these may play a role in the vesicular transport of LOXL1 to the surface membrane of cells [29]. Our study included antigens associated with almost all the genes identified by Dervan et al., but only one such antigen, TMEM9B, showed differentially greater reactivity in diseased patients than in the healthy controls.

In this study, we aimed to identify potential biomarkers specific to XFG using serum samples from 30 patients diagnosed with the condition and 30 healthy controls. In this study, based on adjusted p-values of < 0.05, we identified a total of 7 antigens of interest with FUT2 providing the best ability to discriminate between the groups in our logistic regression model. Most surprising was the ability to build a classification model with relatively high accuracy using the reactivity of only one antigen. Although FUT2 is associated with significant antibody reactivity in the glaucoma patient group, its exact role in the disease’s pathology remains unclear. While FUT2 has been associated with autoimmune diseases such as celiac and Crohn’s [30], its function in these conditions is linked to the secretion of ABO antigens in gastrointestinal mucosa [31], and no direct connection to glaucoma has been established. It is possible that XFG may trigger some immune response which cross-reacts with FUT2 antigens due to molecular mimicry wherein the immune system mistakenly targets the FUT2 proteins while attempting to target a pathological feature of XFG, or that FUT2 antibodies observed in this study may be a marker for other systemic issues more common in XFG patients. One possible direct connection may be that elevated FUT2 is related to a dysfunction within the Schlemm’s canal, a vascular structure associated with glaucoma. The endothelial-mesenchymal transition (EndoMT) has been observed in lymphedema patients, cancer progression, and tissue fibrosis, and it is marked by the loss of endothelial cell properties and their transformation into mesenchymal cells [32]. EndoMT has been implicated in the aging and deterioration of SC, which is highly correlated with glaucoma development [33]. FUT2 has been shown to inhibit EndoMT in colorectal cancer [34], which may indicate that its elevation here in XFG patients may be correlated to the hypothesized increase of EndoMT occurring within the SC. The role of CDH5, a cadherin involved in vascular and lymphatic maintenance [35], in glaucoma could further support this speculation. CDH5 has been identified as a potential biomarker for detecting lymphedema [36] and may also be useful in identifying glaucoma patients. Recent studies have shown a correlation between the coexpression of Smooth Muscle Actin and Platelet Endothelial Cell Adhesion Molecule, known EndoMT markers, and SC aging [37], suggesting a causal relationship between SC deterioration and EndoMT. Furthermore, the downregulation of EndoMT by miR-876-5p [38], which corresponds to an enriched gene set in our study, highlights the possible connection between EndoMT and glaucoma pathology. Understanding the molecular mechanism linking EndoMT and SC could be crucial for deciphering the role of glaucoma-associated genes like FUT2 and CDH5 and their impact on endothelial function. Elucidating the relationship between FUT2, CDH5, and endothelial function in the context of glaucoma may provide valuable insights into the disease’s pathology and open new avenues for targeted therapeutic approaches.

In our analysis, we identified interesting connections among some of the genes associated with the differentially bound proteins, particularly in relation to the expression of miR-24 and their roles in fibrous formations and/or membrane transport. Both LOX and ANXA10 have been shown to be downregulated by increased miR-24 expression [39, 40]. Moreover, ORA using the C2 curated gene set from MSigDB indicated an enrichment in genes associated with collagen fibril formation, most notably *LOX, LOXL2,* and *LOXL3*. These genes have been widely reported to play a role in the pathogenesis of XFG [7, 41]. Furthermore, LOX and other cross-linking proteins are associated with increased tissue stiffness within the eye, particularly in the lamina cribrosa and trabecular meshwork cells [42]. It is also noteworthy that LOX is a target within both the miR-876-5p and miR-3176 regulatory groups identified as enriched in our study. These findings suggest a potential role for miRNAs as regulators of gene expression and their involvement in the molecular mechanisms underlying XFG. In addition to the genes mentioned earlier, *LGSN* and *TMEM9B* also emerged as differentially represented in our study. Although LGSN has been previously identified as differentially expressed in lens tissues derived from rats with cataracts [43], its connection to glaucoma remains unclear. TMEM9B, on the other hand, has not been widely studied in the context of glaucoma, but it is known to be involved in membrane transport processes [44]. It is possible that LGSN and TMEM9B could play roles in fibrous formations and membrane transport within the eye, which may contribute to the development or progression of XFG.

Considering the connections among the genes associated with the differentially bound antigen fragments in our study, including *LOX, ANXA10, LGSN,* and *TMEM9B*, it is important to further investigate their roles in fibrous formation, membrane transport, and their potential regulation by miRNAs. The presence of *LOX1* SNPs, such as rs1050286, rs2165241, rs1048661, and rs3825942, in association with XFG highlights the potential role of genetic variations in the development and progression of this condition. Interestingly, rs1050286 has been shown to alter *LOX-1* expression by modifying miR-24 binding [39], which adds to the growing evidence suggesting a possible connection between miRNA regulation and XFG pathogenesis. The association of rs2165241 and rs1048661 with the progression of XFG in Swedish patients [20] further emphasizes the potential importance of these genetic variations in different populations. Moreover, the identification of rs1048661 and rs3825942 in *LOXL1* as genetic risk factors for XFS and XFG [45], accounting for the majority of XFG cases, indicates a strong link between *LOX1* SNPs and the development of the disease. Taken together, these findings suggest that *LOX1* SNPs could play a significant role in modulating the expression and function of proteins involved in fibrous formation and membrane transport, potentially through the regulation by miRNAs such as miR-24. Further research is needed to unravel the exact mechanisms and implications of these genetic variations in the context of XFG. A better understanding of these relationships could provide valuable insights into the molecular mechanisms underlying exfoliative glaucoma and may ultimately contribute to the development of new therapeutic strategies targeting these pathways in exfoliative glaucoma.

While our primary focus was on markers unique to XFG, we acknowledge the study design’s limitations. These include the potential for capturing associations related to general aspects of glaucoma and the influence of glaucoma medications, which could introduce variations in the autoantibody profile. Additionally, one of the limitations of our study lies in the fact that some of the genes associated with antigens implicated here have no discernible connection to glaucoma or other eye diseases. Most notably fitting that description is DGCR2, which we were unable to find any previous study which connected to any of the other mentioned genes. Also, neither the clustering nor the PCA provided a clear separation of the two groups, which is likely due to the fact that glaucoma is a heterogeneous disease.

Additionally, our study is focused on quantifying the presence of autoantibodies, and many of the interpretations made here assume an effect on the associated proteins. This presents a potential “chicken and egg” scenario: are there more autoantibodies present as a reaction to a higher abundance of the protein, or does the dysfunction of having additional autoantibodies initiate a reaction which compensates and causes adverse effects due to systems attempting to overcome the self-attack? The causality in this relationship remains unclear, and further investigation is required to establish a more comprehensive understanding of the underlying mechanisms.

Another limitation of our study is the sample size. While we had a relatively decent sample size with 30 diseased and 30 healthy individuals, the increasing accessibility to large amounts of biological data highlights the importance of data aggregation and scalable study design for enhanced future investigations. Furthermore, the significant autoantigens identified in this exploratory study need to be further validated by using a new larger cohort to confirm their significance in XFG.

In conclusion, this study suggests several potential biomarkers which may be useful in developing further models of the pathology of XFG. In particular, *CDH5, FUT2*, and the *LOX* family seem to have a relationship that merits additional exploration. Additionally, our results show that the level of antibodies against FUT2 may particularly be useful as a diagnostic biomarker, though these results should be confirmed with additional research.

### Electronic supplementary material

Below is the link to the electronic supplementary material.

**Supplementary Material 1:** List of antigens that showed reactivity against autoantibodies in at least three samples in the preliminary study

**Supplementary Material 2:** Box plots of median fluorescence intensity (MFI) values for differentially bound antigens between healthy controls and exfoliative glaucoma (XFG) patients

**Supplementary Material 3:** English translated patient health survey

## Data Availability

The datasets supporting the conclusions of this article are available in the Github repository, https://github.com/rpotter6298/biomarker_candidates_autoimmunity_profiling_arrays.

## Abbreviations

ANXA10: Annexin A10
BCVA: Best Corrected Visual Acuity
BSA: Bovine Serum Albumin
CDH5: Cadherin 5
EndoMT: Endothelial-Mesenchymal Transition
FC: Fold Change
His6ABP: Hexa-Histidine-tagged Albumin Binding Protein
IgG: Immunoglobulin G
IOP: Intraocular Pressure
LGSN: Lengsin, Lens Protein with Glutamine Synthetase Domain
LOOCV: Leave-One-Out Cross-Validation
LOX: Lysyl Oxidase
LOXL1: Lysyl Oxidase-Like 1
LOXL2: Lysyl Oxidase-Like 2
LOXL3: Lysyl Oxidase-Like 3
MAD: Median Absolute Deviation
MD: Mean Deviation
MFI: Median Fluorescent Intensity
miR or miRNA: MicroRNA
MSigDB: Molecular Signatures Database
ORA: Over-Representation Analysis
PCA: Principal Component Analysis
PBS: Phosphate-Buffered Saline
SD: Standard Deviation
SNPs: Single Nucleotide Polymorphisms
TMEM9B: Transmembrane Protein 9B
wBC: Weighted Box-Cox
XFG: Exfoliation Glaucoma
XFS: Exfoliation Syndrome
