# Supplementary material for: Identification of biomarker candidates for exfoliative glaucoma from autoimmunity profiling
Source: BMC Ophthalmol. 2024 Jan 29;24:44. doi: 10.1186/s12886-024-03314-y (PMC10826272; doi:10.1186/s12886-024-03314-y)
Supplement: Supplementary file 2 — Supplementary Material 2: Box plots of median fluorescence intensity (MFI) values for differentially bound antigens between healthy controls and exfoliative glaucoma (XFG) patients [file 12886_2024_3314_MOESM2_ESM.docx]

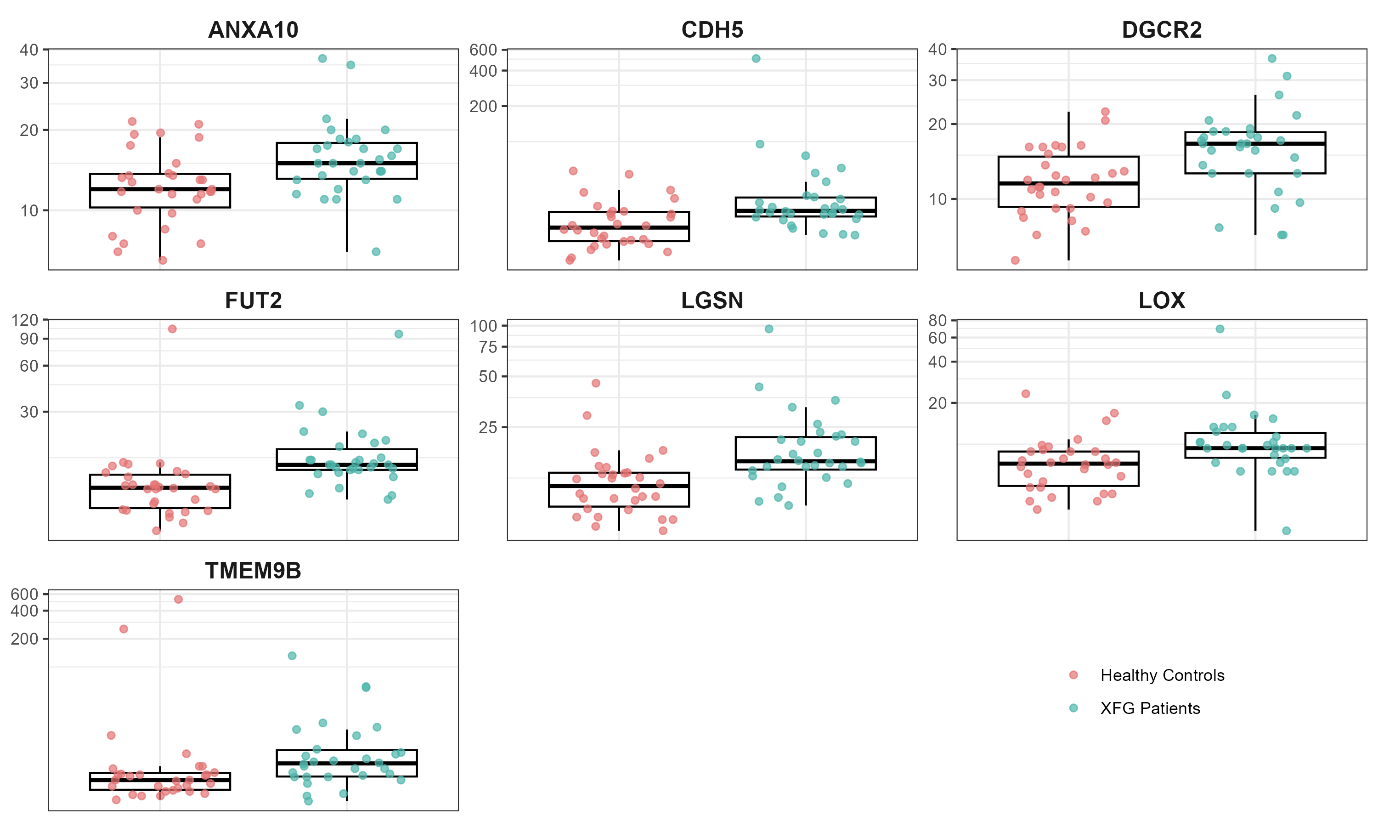


Supplementary Material - Figure 1: Comparative box plots of median fluorescence intensity (MFI) values for antigens with significantly different binding profiles between healthy controls (red) and exfoliative glaucoma (XFG) patients (blue). Y-axes expressed on logarithmic scale.
