# Supplementary material for: Identification of biomarker candidates for exfoliative glaucoma from autoimmunity profiling
Source: BMC Ophthalmol. 2024 Jan 29;24:44. doi: 10.1186/s12886-024-03314-y (PMC10826272; doi:10.1186/s12886-024-03314-y)
Supplement: Supplementary file 3 — Supplementary Material 3: English translated patient health survey [file 12886_2024_3314_MOESM3_ESM.pdf]

Questionnaire- GlauGene

Translation from the original questionnaire (in Swedish).

Please answer the questions below and return the questionnaire to your appointment at the Eye Department. If anything is unclear, please ask the physician or nurse you will meet.

Name and surname:

ID:

1) How long have you known that you have glaucoma? Specify the number of years since you received the diagnosis.

2) Do you have any other eye diseases, or have you been operated on for an eye disease? Specify which other diagnoses/operations you have received and when (year)

3) List any eye drops you are using now.

4) Do you suffer from diabetes?
   YES_____ NO_______
   If you answered "YES", please specify when you received the diagnosis and which treatment are you using.

5) Do you have any autoimmune disease?
   YES___ NO____

6) Are you taking treatment with corticosteroids or any other immunosuppressive treatment?
   YES____ NO ____

7) Do you suffer from migraines?
   YES____ NO____

8) Do you have problems with your memory?
   YES_____ NO_____
   If YES: please specify if you are receiving treatment and which medications you are taking

9) Do you have high blood pressure?
   YES____ NO____
   If YES: specify which medicines you are taking

10) Do you have any other diseases? (non-eye diseases).
    If YES: please specify

11) Do you take any other medicines?
    If YES: please specify.

12) Are you taking vitamin supplements?
    YES _____ NO____
    If YES, please specify which, doses and how long.

13) Smoking is a known risk factor. Have you ever smoked more than 100 cigarettes in your life?
    YES ____ NO_____
14) Have you ever smoked more than once per week?
    YES_____ NO_____

If you answered YES to both questions 13 and 14 please proceed with 15.

15) A) How many cigarettes did you smoke per day?
    B) When did you begin smoking?
    C) When did you quit smoking?
    D) Additional information.

16) Do you know if anyone among your relatives has glaucoma?
    YES____ NO____
    If YES, please specify whom (mother, father, etc).

17) Do you know if anyone among your relatives has any other eye disease?
    YES____ NO____
    If YES, please specify.

18) Ethnic background. As the study aims to detect genes, it's important to know your ethnic background. Genes can vary according to different populations.
    Are both of your parents born in Sweden?
    YES____ NO____
    If NO, please identify which country they are from and when they moved to Sweden (year).

Thanks for your collaboration
